# Bis[bis­(2,2′-bi­pyridine-κ^2^ *N*,*N*′)(carbon­ato-κ^2^ *O*,*O*′)cobalt(III)] 2-{4-[(carboxyl­atometh­yl)carbamo­yl]benz­amido}­acetate hexa­hydrate

**DOI:** 10.1107/S160053681400631X

**Published:** 2014-04-02

**Authors:** Niels-Patrick Pook, Mimoza Gjikaj, Arnold Adam

**Affiliations:** a Institute of Inorganic and Analytical Chemistry, Clausthal University of Technology, Paul-Ernst-Strasse 4, D-38678 Clausthal-Zellerfeld, Germany

## Abstract

The complex cation of the title compound, [Co(CO_3_)(C_10_H_8_N_2_)_2_]_2_(C_12_H_10_N_2_O_6_)·6H_2_O, contains a Co^III^ atom with a distorted octa­hedral coordination environment formed by four N atoms from two bidentate 2,2′-bi­pyridine ligands and one bidentate carbonate anion. The asymmetric unit is completed by one-half of the 2-({4-[(carboxyl­atometh­yl)carbamo­yl]phen­yl}formamido)­acetate dianion, which is located on a centre of inversion, and by three water mol­ecules. Two [Co(CO_3_)(C_10_H_8_N_2_)_2_]^+^ cations are connected through C—H⋯O contacts by the uncoordinating anions. The aromatic rings of the 2,2′-bi­pyridine ligands and di­acetate anions are involved in π–π stacking and C—H⋯π inter­actions. The centroid–centroid distances are in the range 3.4898 (4)–3.6384 (5) Å. The crystal structure is stabilized by further O—H⋯O and N—H⋯O hydrogen bonds, which give rise to a three-dimensional supra­molecular network.

## Related literature   

For related crystal structures of transition metals with 2,2′-(terephthaloylbis(aza­nedi­yl))di­acetate, see: Duan *et al.* (2010 ▶); Kostakis *et al.* (2005 ▶, 2011 ▶); Wisser *et al.* (2008 ▶); Zhang & You (2005 ▶); Zhang *et al.* (2006 ▶). For structures containing the [Co(C_10_H_8_N_2_)_2_(CO_3_)] cation, see: Baca *et al.* (2005 ▶); Lv *et al.* (2007 ▶); Ma *et al.* (2008 ▶); Wojciechowska & Daszkiewicz (2010 ▶). For cds networks, see: Delgado Friedrichs *et al.* (2003 ▶). For π–π and C–H⋯π inter­actions, see: Janiak (2000 ▶); Meyer *et al.* (2003 ▶); Salonen *et al.* (2011 ▶). For coordination polymers including metal-organic frameworks, see: Allendorf *et al.* (2009 ▶); Cook *et al.* (2013 ▶); Schneider (2009 ▶); Yamada *et al.* (2013 ▶). For C—H⋯O hydrogen bonds, see: Desiraju (1991 ▶, 2005 ▶); Steiner (1996 ▶, 1997 ▶). For details of the preparation, see: Cleaver & Pratt (1955 ▶).
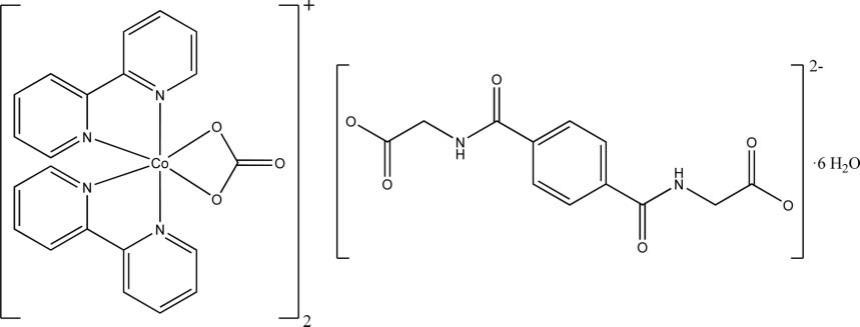


## Experimental   

### 

#### Crystal data  

[Co(CO_3_)(C_10_H_8_N_2_)_2_]_2_(C_12_H_10_N_2_O_6_)·6H_2_O
*M* *_r_* = 1248.94
Triclinic, 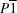

*a* = 10.2198 (13) Å
*b* = 12.1702 (15) Å
*c* = 12.4767 (15) Å
α = 118.119 (9)°
β = 93.936 (10)°
γ = 101.84 (1)°
*V* = 1314.7 (3) Å^3^
*Z* = 1
Mo *K*α radiation
μ = 0.72 mm^−1^
*T* = 223 K
0.22 × 0.21 × 0.20 mm

#### Data collection  

Stoe IPDS 2 diffractometer
Absorption correction: numerical (*X-AREA*; Stoe, 2008) ▶ *T*_min_ = 0.801, *T*_max_ = 0.851
14081 measured reflections
5094 independent reflections
4333 reflections with *I* > 2σ(*I*)
*R*_int_ = 0.088

#### Refinement  

*R*[*F* ^2^ > 2σ(*F* ^2^)] = 0.043
*wR*(*F* ^2^) = 0.106
*S* = 1.05
5094 reflections
487 parameters
All H-atom parameters refined
Δρ_max_ = 0.37 e Å^−3^
Δρ_min_ = −0.71 e Å^−3^

### 

Data collection: *X-AREA* (Stoe, 2008 ▶); cell refinement: *X-AREA*; data reduction: *X-AREA*; program(s) used to solve structure: *SHELXS97* (Sheldrick, 2008 ▶); program(s) used to refine structure: *SHELXL97* (Sheldrick, 2008 ▶); molecular graphics: *DIAMOND* (Brandenburg, 1999 ▶); software used to prepare material for publication: *SHELXL97*, *PLATON* (Spek, 2009 ▶) and *publCIF* (Westrip, 2010 ▶).

## Supplementary Material

Crystal structure: contains datablock(s) I. DOI: 10.1107/S160053681400631X/wm5010sup1.cif

Structure factors: contains datablock(s) I. DOI: 10.1107/S160053681400631X/wm5010Isup2.hkl

Supporting information file. DOI: 10.1107/S160053681400631X/wm5010Isup3.cdx

CCDC reference: 992984

Additional supporting information:  crystallographic information; 3D view; checkCIF report

## Figures and Tables

**Table 1:** Hydrogen-bond geometry (Å, °) *Cg*2 is the centroid of the N2/C7–C11 ring.

*D*—H⋯*A*	*D*—H	H⋯*A*	*D*⋯*A*	*D*—H⋯*A*
O7—H7*A*⋯O6	0.85 (6)	2.11 (6)	2.944 (5)	168 (5)
O7—H7*B*⋯O9	0.79 (7)	2.02 (7)	2.805 (5)	170 (6)
O8—H8*A*⋯O5	0.79 (5)	1.94 (5)	2.722 (4)	170 (5)
O9—H9*A*⋯O4^i^	0.86 (5)	1.98 (6)	2.831 (4)	170 (4)
O9—H9*B*⋯O4	0.76 (5)	2.07 (5)	2.812 (4)	165 (5)
N5—H5*A*⋯O8^ii^	0.74 (4)	2.16 (4)	2.881 (4)	163 (3)
C3—H3⋯O4^iii^	0.94 (3)	2.64 (3)	3.203 (4)	119 (2)
C15—H15⋯O5	0.96 (4)	2.32 (4)	3.264 (4)	165 (3)
C20—H20⋯O6^iv^	0.90 (4)	2.46 (4)	3.162 (4)	135 (3)
C14—H14⋯*Cg*2^v^	0.86 (4)	2.59 (3)	3.419 (3)	160 (3)

Symmetry codes: (i) 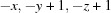; (ii) 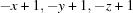; (iii) 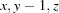; (iv) 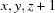; (v) 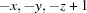.

**Fig. 1.**
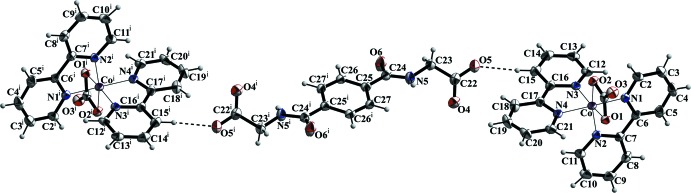
The molecular entities of the title structure with atom labels and displacement ellipsoids of non-H atoms at the 50% probability level. Dashed lines indicate N—H···O and O—H···O hydrogen bonds, as well as C—H···O contacts (see Table 1 for details). [Symmetry code: (i) -x + 1, -y + 2, -z + 1.]

**Fig. 2.**
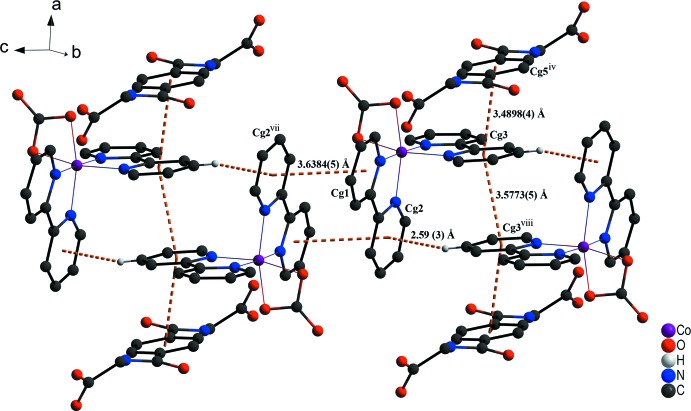
π···π stacking and C–H···π interactions between the aromatic moieties indicated by dashed lines. The hydrogen atoms not involved in interactions have been omitted for clarity. [Symmetry codes: (iv) x, y - 1, z; (vii) -x, -y, -z + 2; (viii) -x, -y, -z + 1.]

**Fig. 3.**
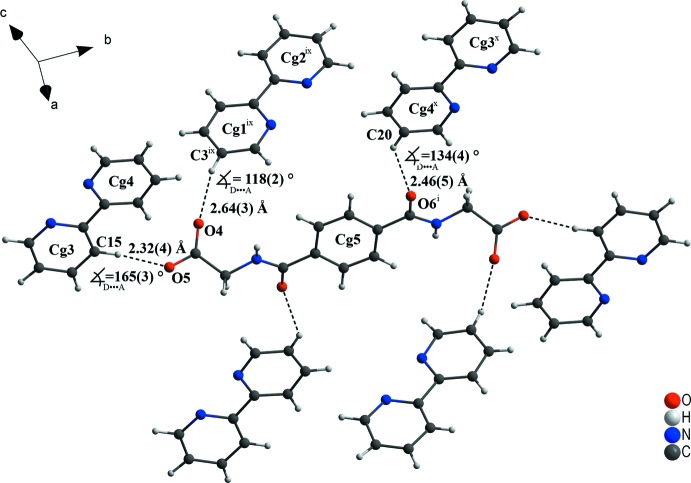
View of the extended network of C—H···O hydrogen bonding with the embedded N,N'-(benzene-1,4-dicarboxamido)diacetate and adjacent phenathrolines. C—H···O contacts are indicated by dashed lines. [Symmetry codes: (i) -x + 1, -y + 2, -z + 1; (ix) x, y + 1, z; (x) -x + 1, -y + 2, -z + 2.]

**Fig. 4.**
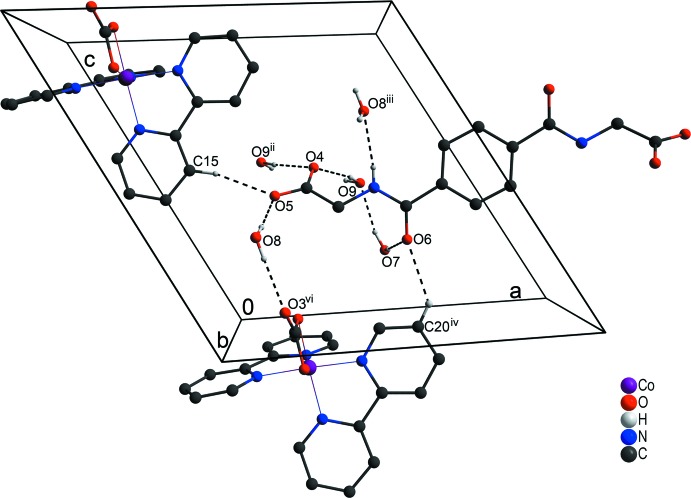
View of the partial unit-cell contents in projection down the b axis with the tree-dimensional hydrogen bonding network. Dashed lines represent the N—H···O, O—H···O and C—H···O hydrogen bonds. Hydrogen atoms not involved in hydrogen bonding have been omitted for clarity. [Symmetry codes: (ii) -x, -y + 1, -z + 1; (iii) -x + 1, -y + 1, z + 1; (vi) x, y, z - 1.]

## supplementary crystallographic information

### 1. Comment

In the past decades, the focus on metal-organic materials including coordination polymers such as metal-organic frameworks and supra­molecules has been expanded rapidly due to their fascinating architectures, their multiple properties as gas storage or luminescent materials as well as their applications in modern metal-organic science, see: Schneider (2009); Allendorf *et al.* (2009); Cook *et al.* (2013); Yamada *et al.* (2013). For the synthesis of such materials the use of different organic linkers with relatively rigid bodies, which contain simultaneously several coordination centers, are required to build up such systems. In the crystal structures of complexes with transition metals and terephthaloylbisglycinate as ligand, zigzag chains are formed, constructing a twofold inter­penetrating cds net (Wisser *et al.*, 2008; Kostakis *et al.*, 2005, 2011; Zhang & You, 2005; Zhang *et al.*, 2006; Duan *et al.*, 2010; Delgado Friedrichs *et al.*, 2003). In our approach we try to substituate one or two of the terephthaloylbisglycinate anions as a bridging linker between two metal coordination centers in the mentioned zigzag chains with bidentate ligands in order to block the coordination on one or more sides of the metal coordination environment, resulting in novel 3D-networks. For this reason we have chosen 2,2'-bi­pyridines as bidentate ligands. As known from literature, nitro­gen-containing aromatic systems exhibit an electron deficity and thus are predestined for π···π-stacking inter­actions among one another and/or with other electron-deficient aromatic systems (Janiak, 2000). Furthermore, with this choice of ligands, the system is offered an alternative route for stabilising the crystal structure (Meyer *et al.* 2003; Salonen *et al.*, 2011).

The asymmetric unit of the title compound, [Co(C_10_H_8_N_2_)_2_(CO_3_)]_2_·(C_12_H_10_O_6_)·6H_2_O, consists of one [Co(C_10_H_8_N_2_)_2_(CO_3_)] cation, half of the terephthaloylbisglycine as counteranion and three water molecules, hold together through a number of noncovalent inter­actions, thereunder hydrogen bonds, C—H···O contacts, π···π- and C—H···π inter­actions (Table 1; Fig. 1). The cation shows a distorted o­cta­hedral coordination sphere defined by two bidentate 2,2'-bi­pyridine ligands and one chelating carbonate anion (Fig. 1). Herein, Co—N and Co—O bond lengths are similar to those found in other bis­(2,2-bi­pyridine-κ^2^N,N)(carbonato-κ^2^O,O)cobalt(III) complexes (Baca *et al.*, 2005; Lv *et al.*, 2007; Ma *et al.*, 2008; Wojciechowska & Daszkiewicz, 2010). The bi­pyridine ligands of two neighbouring complex cations are linked through C—H···π inter­actions. In addition to those inter­actions, the aromatic moities of bi­pyridines and non-coordinating terephthaloylbisglycine are involved in π···π-stacking inter­actions as well as C—H···O contacts (Fig. 2). Figure 3 shows a centered N,N'-(benzene-1,4-dicarboxamido)­diacetate which is embedded in the C—H···O hydrogen bonding network with an adjacent phenathroline ligand. All bond lengths and angles involved in hydrogen bonding are well within the expected ranges (Desiraju, 1991, 2005; Steiner, 1996, 1997). Besides the mentioned non-classical inter­actions, the crystal structure is essentiall stabilised by further hydrogen bonds of the type O—H···O and N—H···O (Tab. 1). A view of the partial unit-cell contents gives an impression of the extended 3-D hydrogen bonding network (Fig. 4).

### 2. Experimental

The starting material, 2,2'-(benzene-1,4-dicarboxamido)­diacetatic acid, was prepared by the method of Cleaver *et al.* (1955). Cesium carbonate (2 mmol), 2,2'-bi­pyridine (1 mmol) and 2,2'-(benzene-1,4-dicarboxamido)­diacetatic acid (1 mmol) were dissolved in a 1:1 mixture of water and methanol (50 ml) and refluxed for 30 minutes. The mixture was allowed to cool to room temperature and an aqueous solution of cobalt nitrate (1 mmol) was slowly added under continuous stirring. The solution changed the color from orange to deep red within one day. Deep red block-shaped crystals of the title compound were obtained by slow evaporation at room temperature. Analysis calculated for C_33_H_32_CoN_6_O_12_: C 51.91, H 4.36, N 11.01%; found: C 51.50, H 4.72, N 11.17%.

### 2.1. Refinement

All hydrogen atoms were located difference Fourier maps and were refined isotropically with no restraints.

### Crystal data

**Table:**

[Co(CO_3_)(C_10_H_8_N_2_)_2_]_2_(C_12_H_10_N_2_O_6_)·6H_2_O	*Z* = 1
*M**_r_* = 1248.94	*F*(000) = 1292
Triclinic, *P*1	*D*_x_ = 1.577 Mg m^−^^3^
Hall symbol: -P 1	Mo *K*α radiation, λ = 0.71073 Å
*a* = 10.2198 (13) Å	Cell parameters from 5094 reflections
*b* = 12.1702 (15) Å	θ = 1.0–26°
*c* = 12.4767 (15) Å	µ = 0.72 mm^−^^1^
α = 118.119 (9)°	*T* = 223 K
β = 93.936 (10)°	Block, dark red
γ = 101.84 (1)°	0.22 × 0.21 × 0.20 mm
*V* = 1314.7 (3) Å^3^	

### Data collection

**Table:**

Stoe IPDS 2 diffractometer	5094 independent reflections
Radiation source: fine-focus sealed tube	4333 reflections with *I* > 2σ(*I*)
Graphite monochromator	*R*_int_ = 0.088
ω scans	θ_max_ = 26.0°, θ_min_ = 2.1°
Absorption correction: numerical (*X-AREA*; Stoe, 2008)	*h* = −12→12
*T*_min_ = 0.801, *T*_max_ = 0.851	*k* = −15→15
14081 measured reflections	*l* = −15→15

### Refinement

**Table:**

Refinement on *F*^2^	Primary atom site location: structure-invariant direct methods
Least-squares matrix: full	Secondary atom site location: difference Fourier map
*R*[*F*^2^ > 2σ(*F*^2^)] = 0.043	Hydrogen site location: inferred from neighbouring sites
*wR*(*F*^2^) = 0.106	All H-atom parameters refined
*S* = 1.05	*w* = 1/[σ^2^(*F*_o_^2^) + (0.0507*P*)^2^ + 0.6944*P*] where *P* = (*F*_o_^2^ + 2*F*_c_^2^)/3
5094 reflections	(Δ/σ)_max_ = 0.001
487 parameters	Δρ_max_ = 0.37 e Å^−^^3^
0 restraints	Δρ_min_ = −0.71 e Å^−^^3^

### Special details

**Table:**

Geometry. All e.s.d.'s (except the e.s.d. in the dihedral angle between two l.s. planes) are estimated using the full covariance matrix. The cell e.s.d.'s are taken into account individually in the estimation of e.s.d.'s in distances, angles and torsion angles; correlations between e.s.d.'s in cell parameters are only used when they are defined by crystal symmetry. An approximate (isotropic) treatment of cell e.s.d.'s is used for estimating e.s.d.'s involving l.s. planes.
Refinement. Refinement of *F*^2^ against ALL reflections. The weighted *R*-factor *wR* and goodness of fit *S* are based on *F*^2^, conventional *R*-factors *R* are based on *F*, with *F* set to zero for negative *F*^2^. The threshold expression of *F*^2^ > σ(*F*^2^) is used only for calculating *R*-factors(gt) *etc*. and is not relevant to the choice of reflections for refinement. *R*-factors based on *F*^2^ are statistically about twice as large as those based on *F*, and *R*-factors based on ALL data will be even larger.

### Fractional atomic coordinates and isotropic or equivalent isotropic displacement parameters (Å^2^)

**Table:**

	*x*	*y*	*z*	*U*_iso_*/*U*_eq_	
Co	0.21576 (3)	0.13013 (3)	0.85140 (3)	0.01767 (11)	
O1	0.28625 (18)	0.19311 (17)	1.02079 (16)	0.0237 (4)	
O2	0.40218 (17)	0.13060 (17)	0.87431 (16)	0.0247 (4)	
O3	0.50087 (19)	0.18433 (19)	1.06633 (18)	0.0319 (4)	
O4	0.2036 (2)	0.5200 (2)	0.5181 (2)	0.0471 (6)	
O5	0.2900 (2)	0.3522 (2)	0.4300 (2)	0.0463 (6)	
O6	0.3510 (3)	0.6605 (2)	0.26307 (19)	0.0431 (5)	
O7	0.0512 (4)	0.5777 (3)	0.2127 (4)	0.0641 (8)	
H7A	0.137 (6)	0.590 (5)	0.221 (5)	0.078 (17)*	
H7B	0.041 (6)	0.584 (6)	0.277 (6)	0.10 (2)*	
O8	0.4733 (3)	0.2331 (3)	0.3056 (3)	0.0498 (6)	
H8A	0.414 (5)	0.263 (4)	0.334 (4)	0.060 (13)*	
H8B	0.475 (4)	0.218 (4)	0.237 (4)	0.042 (10)*	
O9	0.0237 (3)	0.6356 (3)	0.4544 (3)	0.0517 (7)	
H9A	−0.049 (5)	0.597 (5)	0.466 (5)	0.075 (15)*	
H9B	0.082 (6)	0.617 (5)	0.478 (5)	0.078 (17)*	
N1	0.1557 (2)	−0.04188 (19)	0.82682 (19)	0.0211 (4)	
N2	0.0283 (2)	0.1307 (2)	0.86728 (19)	0.0212 (4)	
N3	0.1847 (2)	0.07228 (19)	0.67539 (19)	0.0198 (4)	
N4	0.2688 (2)	0.2999 (2)	0.8673 (2)	0.0214 (4)	
N5	0.3909 (3)	0.6596 (2)	0.4424 (3)	0.0326 (5)	
H5A	0.414 (3)	0.695 (3)	0.510 (3)	0.031 (9)*	
C1	0.4045 (2)	0.1708 (2)	0.9927 (2)	0.0222 (5)	
C2	0.2320 (3)	−0.1265 (3)	0.8002 (3)	0.0271 (5)	
H2	0.320 (3)	−0.095 (3)	0.794 (3)	0.019 (7)*	
C3	0.1780 (3)	−0.2506 (3)	0.7780 (3)	0.0338 (6)	
H3	0.234 (3)	−0.308 (3)	0.755 (3)	0.033 (8)*	
C4	0.0441 (3)	−0.2879 (3)	0.7877 (3)	0.0365 (7)	
H4	0.010 (3)	−0.366 (3)	0.780 (3)	0.035 (8)*	
C5	−0.0343 (3)	−0.2007 (3)	0.8171 (3)	0.0309 (6)	
H5	−0.124 (4)	−0.225 (3)	0.825 (3)	0.036 (9)*	
C6	0.0243 (2)	−0.0784 (2)	0.8355 (2)	0.0211 (5)	
C7	−0.0479 (2)	0.0219 (2)	0.8617 (2)	0.0208 (5)	
C8	−0.1824 (3)	0.0089 (3)	0.8773 (2)	0.0280 (5)	
H8	−0.229 (3)	−0.064 (3)	0.875 (3)	0.019 (7)*	
C9	−0.2409 (3)	0.1084 (3)	0.8980 (3)	0.0356 (7)	
H9	−0.334 (3)	0.099 (3)	0.909 (3)	0.036 (8)*	
C10	−0.1639 (3)	0.2174 (3)	0.9019 (3)	0.0372 (7)	
H10	−0.200 (3)	0.285 (3)	0.908 (3)	0.037 (9)*	
C11	−0.0297 (3)	0.2267 (3)	0.8866 (3)	0.0283 (6)	
H11	0.026 (3)	0.304 (3)	0.890 (3)	0.030 (8)*	
C12	0.1403 (3)	−0.0507 (2)	0.5811 (2)	0.0250 (5)	
H12	0.115 (3)	−0.121 (3)	0.602 (3)	0.024 (7)*	
C13	0.1368 (3)	−0.0807 (3)	0.4597 (2)	0.0289 (6)	
H13	0.102 (3)	−0.177 (3)	0.392 (3)	0.035 (8)*	
C14	0.1773 (3)	0.0183 (3)	0.4329 (3)	0.0323 (6)	
H14	0.177 (3)	−0.001 (3)	0.357 (3)	0.030 (8)*	
C15	0.2186 (3)	0.1458 (3)	0.5287 (3)	0.0289 (6)	
H15	0.250 (3)	0.217 (3)	0.514 (3)	0.038 (9)*	
C16	0.2210 (2)	0.1699 (2)	0.6487 (2)	0.0214 (5)	
C17	0.2658 (2)	0.2992 (2)	0.7585 (2)	0.0216 (5)	
C18	0.3014 (3)	0.4133 (3)	0.7543 (3)	0.0325 (6)	
H18	0.295 (4)	0.408 (4)	0.675 (4)	0.056 (11)*	
C19	0.3429 (3)	0.5296 (3)	0.8646 (3)	0.0375 (7)	
H19	0.372 (3)	0.613 (3)	0.871 (3)	0.038 (9)*	
C20	0.3474 (3)	0.5293 (3)	0.9753 (3)	0.0320 (6)	
H20	0.372 (4)	0.604 (4)	1.049 (4)	0.046 (10)*	
C21	0.3088 (3)	0.4127 (2)	0.9739 (3)	0.0262 (5)	
H21	0.310 (3)	0.410 (3)	1.045 (3)	0.024 (7)*	
C22	0.2783 (3)	0.4602 (3)	0.4507 (3)	0.0308 (6)	
C23	0.3609 (4)	0.5196 (3)	0.3847 (3)	0.0370 (7)	
H23A	0.445 (5)	0.497 (4)	0.378 (4)	0.067 (13)*	
H23B	0.313 (4)	0.484 (4)	0.299 (4)	0.046 (10)*	
C24	0.3921 (3)	0.7198 (3)	0.3761 (3)	0.0280 (6)	
C25	0.4476 (2)	0.8647 (3)	0.4443 (2)	0.0251 (5)	
C26	0.4791 (3)	0.9241 (3)	0.3728 (3)	0.0266 (5)	
H26	0.471 (3)	0.873 (3)	0.288 (3)	0.026 (7)*	
C27	0.4691 (3)	0.9436 (3)	0.5722 (3)	0.0270 (5)	
H27	0.449 (3)	0.902 (3)	0.620 (3)	0.032 (8)*	

### Atomic displacement parameters (Å^2^)

**Table:**

	*U*^11^	*U*^22^	*U*^33^	*U*^12^	*U*^13^	*U*^23^
Co	0.01697 (17)	0.01872 (17)	0.01930 (18)	0.00522 (12)	0.00537 (12)	0.01058 (13)
O1	0.0240 (9)	0.0276 (9)	0.0224 (9)	0.0086 (7)	0.0069 (7)	0.0138 (7)
O2	0.0211 (8)	0.0298 (9)	0.0252 (9)	0.0072 (7)	0.0075 (7)	0.0148 (8)
O3	0.0260 (9)	0.0385 (11)	0.0305 (10)	0.0080 (8)	0.0008 (8)	0.0175 (9)
O4	0.0482 (13)	0.0428 (12)	0.0563 (15)	0.0148 (10)	0.0271 (11)	0.0263 (12)
O5	0.0538 (14)	0.0439 (13)	0.0660 (16)	0.0205 (11)	0.0268 (12)	0.0418 (12)
O6	0.0620 (15)	0.0367 (11)	0.0270 (11)	0.0028 (10)	0.0031 (10)	0.0179 (9)
O7	0.064 (2)	0.0615 (18)	0.076 (2)	0.0043 (15)	0.0015 (16)	0.0475 (17)
O8	0.0623 (17)	0.0619 (16)	0.0335 (13)	0.0327 (14)	0.0063 (12)	0.0242 (12)
O9	0.0511 (15)	0.0494 (15)	0.0754 (19)	0.0170 (13)	0.0267 (14)	0.0441 (15)
N1	0.0235 (10)	0.0216 (10)	0.0206 (10)	0.0064 (8)	0.0048 (8)	0.0122 (8)
N2	0.0221 (10)	0.0254 (10)	0.0196 (10)	0.0084 (8)	0.0054 (8)	0.0130 (9)
N3	0.0179 (9)	0.0220 (10)	0.0213 (10)	0.0070 (8)	0.0060 (8)	0.0112 (8)
N4	0.0198 (10)	0.0228 (10)	0.0247 (11)	0.0060 (8)	0.0068 (8)	0.0137 (9)
N5	0.0426 (14)	0.0283 (12)	0.0278 (14)	0.0033 (10)	0.0044 (11)	0.0173 (11)
C1	0.0216 (11)	0.0218 (11)	0.0244 (12)	0.0023 (9)	0.0034 (9)	0.0138 (10)
C2	0.0274 (13)	0.0279 (13)	0.0279 (13)	0.0132 (11)	0.0059 (10)	0.0131 (11)
C3	0.0429 (16)	0.0256 (13)	0.0337 (15)	0.0145 (12)	0.0043 (12)	0.0138 (12)
C4	0.0481 (18)	0.0186 (13)	0.0374 (16)	0.0038 (12)	0.0003 (13)	0.0127 (12)
C5	0.0328 (15)	0.0251 (13)	0.0301 (14)	0.0007 (11)	0.0027 (11)	0.0134 (11)
C6	0.0212 (11)	0.0218 (11)	0.0184 (11)	0.0028 (9)	0.0028 (9)	0.0098 (10)
C7	0.0195 (11)	0.0254 (12)	0.0163 (11)	0.0046 (9)	0.0018 (9)	0.0102 (10)
C8	0.0216 (12)	0.0395 (15)	0.0276 (13)	0.0036 (11)	0.0054 (10)	0.0221 (12)
C9	0.0240 (13)	0.0542 (18)	0.0369 (16)	0.0172 (13)	0.0121 (11)	0.0256 (14)
C10	0.0311 (15)	0.0459 (17)	0.0451 (17)	0.0240 (13)	0.0148 (13)	0.0244 (15)
C11	0.0277 (13)	0.0278 (13)	0.0349 (15)	0.0129 (11)	0.0104 (11)	0.0173 (12)
C12	0.0227 (12)	0.0233 (12)	0.0231 (13)	0.0071 (10)	0.0026 (10)	0.0069 (10)
C13	0.0276 (13)	0.0323 (14)	0.0210 (12)	0.0117 (11)	0.0027 (10)	0.0076 (11)
C14	0.0282 (14)	0.0476 (17)	0.0211 (13)	0.0135 (12)	0.0050 (10)	0.0158 (13)
C15	0.0274 (13)	0.0364 (15)	0.0269 (13)	0.0076 (11)	0.0064 (10)	0.0190 (12)
C16	0.0159 (10)	0.0256 (12)	0.0249 (12)	0.0044 (9)	0.0036 (9)	0.0148 (10)
C17	0.0181 (11)	0.0243 (12)	0.0249 (12)	0.0049 (9)	0.0044 (9)	0.0144 (11)
C18	0.0312 (14)	0.0308 (14)	0.0390 (16)	0.0027 (11)	0.0022 (12)	0.0230 (13)
C19	0.0367 (15)	0.0229 (14)	0.0501 (19)	0.0041 (12)	−0.0023 (13)	0.0190 (13)
C20	0.0271 (13)	0.0227 (13)	0.0381 (16)	0.0068 (11)	−0.0011 (11)	0.0097 (12)
C21	0.0258 (13)	0.0242 (12)	0.0255 (13)	0.0075 (10)	0.0045 (10)	0.0097 (11)
C22	0.0295 (13)	0.0341 (14)	0.0310 (14)	0.0044 (11)	0.0036 (11)	0.0199 (12)
C23	0.0492 (18)	0.0299 (15)	0.0372 (17)	0.0089 (13)	0.0161 (14)	0.0206 (13)
C24	0.0259 (13)	0.0342 (14)	0.0279 (14)	0.0101 (11)	0.0087 (10)	0.0172 (12)
C25	0.0205 (11)	0.0331 (13)	0.0287 (13)	0.0107 (10)	0.0072 (10)	0.0191 (11)
C26	0.0256 (13)	0.0339 (14)	0.0247 (13)	0.0107 (11)	0.0087 (10)	0.0165 (11)
C27	0.0280 (13)	0.0354 (14)	0.0282 (13)	0.0108 (11)	0.0115 (10)	0.0224 (12)

### Geometric parameters (Å, º)

**Table:**

Co—O1	1.9002 (18)	C7—C8	1.389 (3)
Co—O2	1.9054 (17)	C8—C9	1.384 (4)
Co—N1	1.922 (2)	C8—H8	0.91 (3)
Co—N4	1.934 (2)	C9—C10	1.373 (5)
Co—N2	1.940 (2)	C9—H9	0.97 (3)
Co—N3	1.943 (2)	C10—C11	1.387 (4)
Co—C1	2.322 (3)	C10—H10	0.94 (3)
O1—C1	1.326 (3)	C11—H11	0.97 (3)
O2—C1	1.316 (3)	C12—C13	1.377 (4)
O3—C1	1.228 (3)	C12—H12	0.99 (3)
O4—C22	1.248 (4)	C13—C14	1.388 (4)
O5—C22	1.248 (4)	C13—H13	1.04 (3)
O6—C24	1.234 (3)	C14—C15	1.389 (4)
O7—H7A	0.85 (6)	C14—H14	0.87 (3)
O7—H7B	0.79 (7)	C15—C16	1.380 (4)
O8—H8A	0.79 (5)	C15—H15	0.96 (4)
O8—H8B	0.79 (4)	C16—C17	1.466 (3)
O9—H9A	0.86 (5)	C17—C18	1.388 (4)
O9—H9B	0.76 (5)	C18—C19	1.383 (4)
N1—C2	1.349 (3)	C18—H18	0.95 (4)
N1—C6	1.354 (3)	C19—C20	1.381 (5)
N2—C11	1.347 (3)	C19—H19	0.95 (4)
N2—C7	1.359 (3)	C20—C21	1.383 (4)
N3—C12	1.348 (3)	C20—H20	0.90 (4)
N3—C16	1.368 (3)	C21—H21	0.91 (3)
N4—C21	1.340 (3)	C22—O5	1.248 (4)
N4—C17	1.352 (3)	C22—O4	1.248 (4)
N5—C24	1.338 (4)	C22—C23	1.523 (4)
N5—C23	1.454 (4)	C23—H23A	0.95 (4)
N5—H5A	0.74 (4)	C23—H23B	0.98 (4)
C2—C3	1.380 (4)	C24—O6	1.234 (3)
C2—H2	0.93 (3)	C24—C25	1.505 (4)
C3—C4	1.384 (5)	C25—C27	1.391 (4)
C3—H3	0.94 (3)	C25—C26	1.405 (4)
C4—C5	1.388 (4)	C26—C27^i^	1.380 (4)
C4—H4	0.90 (4)	C26—H26	0.92 (3)
C5—C6	1.385 (4)	C27—C26^i^	1.380 (4)
C5—H5	0.94 (4)	C27—H27	0.95 (3)
C6—C7	1.473 (3)		
			
O1—Co—O2	69.28 (8)	C10—C9—C8	118.7 (3)
O1—Co—N1	89.82 (8)	C10—C9—H9	122.4 (19)
O2—Co—N1	91.79 (8)	C8—C9—H9	119 (2)
O1—Co—N4	93.35 (8)	C9—C10—C11	120.0 (3)
O2—Co—N4	90.33 (8)	C9—C10—H10	123 (2)
N1—Co—N4	176.66 (9)	C11—C10—H10	117 (2)
O1—Co—N2	97.26 (8)	N2—C11—C10	121.6 (3)
O2—Co—N2	165.72 (8)	N2—C11—H11	118.0 (18)
N1—Co—N2	83.18 (9)	C10—C11—H11	120.4 (18)
N4—Co—N2	95.35 (9)	N3—C12—C13	121.8 (3)
O1—Co—N3	167.67 (8)	N3—C12—H12	118.0 (17)
O2—Co—N3	98.81 (8)	C13—C12—H12	120.1 (17)
N1—Co—N3	93.85 (9)	C12—C13—C14	119.3 (3)
N4—Co—N3	83.28 (9)	C12—C13—H13	117.8 (19)
N2—Co—N3	94.86 (8)	C14—C13—H13	122.9 (19)
O1—Co—C1	34.83 (8)	C13—C14—C15	119.5 (3)
O2—Co—C1	34.52 (8)	C13—C14—H14	119 (2)
N1—Co—C1	89.28 (9)	C15—C14—H14	121 (2)
N4—Co—C1	93.93 (9)	C16—C15—C14	118.6 (3)
N2—Co—C1	131.67 (9)	C16—C15—H15	119 (2)
N3—Co—C1	133.34 (8)	C14—C15—H15	122 (2)
C1—O1—Co	90.26 (14)	N3—C16—C15	122.0 (2)
C1—O2—Co	90.34 (14)	N3—C16—C17	113.7 (2)
H7A—O7—H7B	100 (5)	C15—C16—C17	124.3 (2)
H8A—O8—H8B	115 (4)	N4—C17—C18	121.5 (2)
H9A—O9—H9B	106 (5)	N4—C17—C16	114.4 (2)
C2—N1—C6	119.2 (2)	C18—C17—C16	124.1 (2)
C2—N1—Co	125.61 (19)	C19—C18—C17	118.7 (3)
C6—N1—Co	115.20 (16)	C19—C18—H18	123 (2)
C11—N2—C7	118.7 (2)	C17—C18—H18	118 (2)
C11—N2—Co	127.23 (18)	C20—C19—C18	119.4 (3)
C7—N2—Co	114.07 (16)	C20—C19—H19	116 (2)
C12—N3—C16	118.7 (2)	C18—C19—H19	125 (2)
C12—N3—Co	127.23 (18)	C19—C20—C21	119.4 (3)
C16—N3—Co	113.90 (16)	C19—C20—H20	122 (2)
C21—N4—C17	119.6 (2)	C21—C20—H20	119 (2)
C21—N4—Co	125.91 (19)	N4—C21—C20	121.4 (3)
C17—N4—Co	114.51 (16)	N4—C21—H21	117.4 (19)
C24—N5—C23	122.3 (3)	C20—C21—H21	121.2 (19)
C24—N5—H5A	123 (3)	O5—C22—O4	125.3 (3)
C23—N5—H5A	114 (3)	O5—C22—O4	125.3 (3)
O3—C1—O2	125.3 (2)	O5—C22—O4	125.3 (3)
O3—C1—O1	124.8 (2)	O5—C22—O4	125.3 (3)
O2—C1—O1	109.9 (2)	O5—C22—C23	115.8 (3)
O3—C1—Co	175.78 (19)	O5—C22—C23	115.8 (3)
O2—C1—Co	55.14 (11)	O4—C22—C23	119.0 (3)
O1—C1—Co	54.91 (12)	O4—C22—C23	119.0 (3)
N1—C2—C3	121.5 (3)	N5—C23—C22	115.3 (3)
N1—C2—H2	114.3 (17)	N5—C23—H23A	108 (3)
C3—C2—H2	124.1 (17)	C22—C23—H23A	110 (3)
C2—C3—C4	119.4 (3)	N5—C23—H23B	107 (2)
C2—C3—H3	118 (2)	C22—C23—H23B	111 (2)
C4—C3—H3	122 (2)	H23A—C23—H23B	106 (3)
C3—C4—C5	119.4 (3)	O6—C24—N5	122.2 (3)
C3—C4—H4	120 (2)	O6—C24—N5	122.2 (3)
C5—C4—H4	120 (2)	O6—C24—C25	120.2 (3)
C6—C5—C4	118.7 (3)	O6—C24—C25	120.2 (3)
C6—C5—H5	122 (2)	N5—C24—C25	117.5 (2)
C4—C5—H5	120 (2)	C27—C25—C26	118.0 (3)
N1—C6—C5	121.8 (2)	C27—C25—C24	124.9 (2)
N1—C6—C7	113.5 (2)	C26—C25—C24	117.1 (2)
C5—C6—C7	124.7 (2)	C27^i^—C26—C25	121.0 (3)
N2—C7—C8	121.6 (2)	C27^i^—C26—H26	119 (2)
N2—C7—C6	113.9 (2)	C25—C26—H26	119 (2)
C8—C7—C6	124.5 (2)	C26^i^—C27—C25	121.1 (3)
C9—C8—C7	119.4 (3)	C26^i^—C27—H27	121.7 (19)
C9—C8—H8	121.6 (17)	C25—C27—H27	117.2 (19)
C7—C8—H8	119.0 (17)		
			
O2—Co—O1—C1	−2.99 (13)	C2—N1—C6—C5	−0.4 (4)
N1—Co—O1—C1	89.00 (14)	Co—N1—C6—C5	178.4 (2)
N4—Co—O1—C1	−92.08 (14)	C2—N1—C6—C7	−178.5 (2)
N2—Co—O1—C1	172.09 (14)	Co—N1—C6—C7	0.3 (3)
N3—Co—O1—C1	−18.4 (4)	C4—C5—C6—N1	−0.7 (4)
O1—Co—O2—C1	3.01 (13)	C4—C5—C6—C7	177.2 (3)
N1—Co—O2—C1	−86.12 (14)	C11—N2—C7—C8	−1.0 (4)
N4—Co—O2—C1	96.46 (14)	Co—N2—C7—C8	176.95 (19)
N2—Co—O2—C1	−17.2 (4)	C11—N2—C7—C6	177.8 (2)
N3—Co—O2—C1	179.71 (14)	Co—N2—C7—C6	−4.3 (3)
O1—Co—N1—C2	−86.1 (2)	N1—C6—C7—N2	2.6 (3)
O2—Co—N1—C2	−16.8 (2)	C5—C6—C7—N2	−175.4 (2)
N2—Co—N1—C2	176.6 (2)	N1—C6—C7—C8	−178.7 (2)
N3—Co—N1—C2	82.2 (2)	C5—C6—C7—C8	3.3 (4)
C1—Co—N1—C2	−51.2 (2)	N2—C7—C8—C9	0.5 (4)
O1—Co—N1—C6	95.23 (18)	C6—C7—C8—C9	−178.1 (3)
O2—Co—N1—C6	164.50 (18)	C7—C8—C9—C10	0.3 (4)
N2—Co—N1—C6	−2.10 (17)	C8—C9—C10—C11	−0.7 (5)
N3—Co—N1—C6	−96.54 (18)	C7—N2—C11—C10	0.6 (4)
C1—Co—N1—C6	130.06 (18)	Co—N2—C11—C10	−177.0 (2)
O1—Co—N2—C11	92.3 (2)	C9—C10—C11—N2	0.2 (5)
O2—Co—N2—C11	111.3 (4)	C16—N3—C12—C13	3.1 (3)
N1—Co—N2—C11	−178.7 (2)	Co—N3—C12—C13	−172.50 (18)
N4—Co—N2—C11	−1.7 (2)	N3—C12—C13—C14	−1.3 (4)
N3—Co—N2—C11	−85.4 (2)	C12—C13—C14—C15	−1.0 (4)
C1—Co—N2—C11	98.4 (2)	C13—C14—C15—C16	1.5 (4)
O1—Co—N2—C7	−85.37 (17)	C12—N3—C16—C15	−2.6 (3)
O2—Co—N2—C7	−66.4 (4)	Co—N3—C16—C15	173.61 (19)
N1—Co—N2—C7	3.57 (17)	C12—N3—C16—C17	179.2 (2)
N4—Co—N2—C7	−179.45 (17)	Co—N3—C16—C17	−4.6 (2)
N3—Co—N2—C7	96.87 (17)	C14—C15—C16—N3	0.3 (4)
C1—Co—N2—C7	−79.34 (19)	C14—C15—C16—C17	178.3 (2)
O1—Co—N3—C12	105.1 (4)	C21—N4—C17—C18	−1.0 (4)
O2—Co—N3—C12	90.5 (2)	Co—N4—C17—C18	−179.89 (19)
N1—Co—N3—C12	−1.9 (2)	C21—N4—C17—C16	179.7 (2)
N4—Co—N3—C12	179.8 (2)	Co—N4—C17—C16	0.8 (3)
N2—Co—N3—C12	−85.4 (2)	N3—C16—C17—N4	2.5 (3)
C1—Co—N3—C12	90.7 (2)	C15—C16—C17—N4	−175.6 (2)
O1—Co—N3—C16	−70.7 (4)	N3—C16—C17—C18	−176.8 (2)
O2—Co—N3—C16	−85.28 (16)	C15—C16—C17—C18	5.1 (4)
N1—Co—N3—C16	−177.70 (16)	N4—C17—C18—C19	1.2 (4)
N4—Co—N3—C16	4.01 (16)	C16—C17—C18—C19	−179.6 (2)
N2—Co—N3—C16	98.84 (16)	C17—C18—C19—C20	−0.3 (4)
C1—Co—N3—C16	−85.06 (18)	C18—C19—C20—C21	−0.7 (4)
O1—Co—N4—C21	−13.4 (2)	C17—N4—C21—C20	−0.1 (4)
O2—Co—N4—C21	−82.6 (2)	Co—N4—C21—C20	178.68 (19)
N2—Co—N4—C21	84.3 (2)	C19—C20—C21—N4	1.0 (4)
N3—Co—N4—C21	178.5 (2)	O5—O5—C22—O4	0.0 (8)
C1—Co—N4—C21	−48.2 (2)	O5—O5—C22—O4	0.0 (8)
O1—Co—N4—C17	165.49 (16)	O5—O5—C22—C23	0.0 (8)
O2—Co—N4—C17	96.23 (17)	O4—O4—C22—O5	0.0 (3)
N2—Co—N4—C17	−96.89 (17)	O4—O4—C22—O5	0.0 (3)
N3—Co—N4—C17	−2.61 (16)	O4—O4—C22—C23	0.0 (5)
C1—Co—N4—C17	130.59 (17)	C24—N5—C23—C22	−142.9 (3)
Co—O2—C1—O3	174.9 (2)	O5—C22—C23—N5	−156.8 (3)
Co—O2—C1—O1	−4.29 (19)	O5—C22—C23—N5	−156.8 (3)
Co—O1—C1—O3	−174.9 (2)	O4—C22—C23—N5	24.5 (4)
Co—O1—C1—O2	4.30 (19)	O4—C22—C23—N5	24.5 (4)
O1—Co—C1—O2	−175.1 (2)	O6—O6—C24—N5	0.0 (2)
N1—Co—C1—O2	94.21 (14)	O6—O6—C24—C25	0.0 (3)
N4—Co—C1—O2	−84.86 (14)	C23—N5—C24—O6	9.6 (4)
N2—Co—C1—O2	174.41 (13)	C23—N5—C24—O6	9.6 (4)
N3—Co—C1—O2	−0.40 (19)	C23—N5—C24—C25	−169.8 (3)
O2—Co—C1—O1	175.1 (2)	O6—C24—C25—C27	165.5 (3)
N1—Co—C1—O1	−90.72 (14)	O6—C24—C25—C27	165.5 (3)
N4—Co—C1—O1	90.21 (14)	N5—C24—C25—C27	−15.1 (4)
N2—Co—C1—O1	−10.53 (18)	O6—C24—C25—C26	−14.2 (4)
N3—Co—C1—O1	174.67 (13)	O6—C24—C25—C26	−14.2 (4)
C6—N1—C2—C3	1.9 (4)	N5—C24—C25—C26	165.1 (2)
Co—N1—C2—C3	−176.7 (2)	C27—C25—C26—C27^i^	0.5 (4)
N1—C2—C3—C4	−2.4 (4)	C24—C25—C26—C27^i^	−179.8 (2)
C2—C3—C4—C5	1.3 (5)	C26—C25—C27—C26^i^	−0.5 (4)
C3—C4—C5—C6	0.2 (4)	C24—C25—C27—C26^i^	179.8 (2)

Symmetry code: (i) −*x*+1, −*y*+2, −*z*+1.

### Hydrogen-bond geometry (Å, º)

Cg2 is the centroid of the N2/C7–C11 ring.

**Table:**

*D*—H···*A*	*D*—H	H···*A*	*D*···*A*	*D*—H···*A*
O7—H7*A*···O6	0.85 (6)	2.11 (6)	2.944 (5)	168 (5)
O7—H7*B*···O9	0.79 (7)	2.02 (7)	2.805 (5)	170 (6)
O8—H8*A*···O5	0.79 (5)	1.94 (5)	2.722 (4)	170 (5)
O9—H9*A*···O4^ii^	0.86 (5)	1.98 (6)	2.831 (4)	170 (4)
O9—H9*B*···O4	0.76 (5)	2.07 (5)	2.812 (4)	165 (5)
N5—H5*A*···O8^iii^	0.74 (4)	2.16 (4)	2.881 (4)	163 (3)
C3—H3···O4^iv^	0.94 (3)	2.64 (3)	3.203 (4)	119 (2)
C15—H15···O5	0.96 (4)	2.32 (4)	3.264 (4)	165 (3)
C20—H20···O6^v^	0.90 (4)	2.46 (4)	3.162 (4)	135 (3)
C14—H14···*Cg*2^vi^	0.86 (4)	2.59 (3)	3.419 (3)	160 (3)

Symmetry codes: (ii) −*x*, −*y*+1, −*z*+1; (iii) −*x*+1, −*y*+1, −*z*+1; (iv) *x*, *y*−1, *z*; (v) *x*, *y*, *z*+1; (vi) −*x*, −*y*, −*z*+1.

### C—H···π interactions (Å, °)

*Cg*2 is the centroid of the N2/C7–C11 ring.

**Table:**

D—H···*Cg*	D—H	H···*Cg*	D—H···*Cg*	D—H···*Cg*
C14^viii^—H14^viii^···*Cg*2	0.86 (4)	2.59 (3)	3.4191 (34)	160 (3)

Symmetry codes: (viii) -x, -y, -z + 1.
